# Treatment Patterns for Patients on Overactive Bladder Therapy: A Retrospective Statistical Analysis Using Canadian Claims Data

**DOI:** 10.36469/9841

**Published:** 2016-10-28

**Authors:** Adrian Wagg, Demitri Diles, Todd Berner

**Affiliations:** 1 Geriatric Medicine, Department of Medicine University of Alberta, Edmonton, Alberta, Canada; 2 Patient Access and Government Relations, Astellas Pharma Canada, Inc., Markham, Ontario, Canada; 3 Formerly of Astellas Scientific & Medical Affairs, Inc., Northbrook, IL, USA

**Keywords:** switching patterns, antimuscarinic drugs, discontinuation, persistence, overactive bladder

## Abstract

**Background:** Overactive bladder (OAB) is a chronic condition which may be associated with a significant negative impact on quality of life. Antimuscarinic drugs are currently the mainstay of medical therapy, but persistence and adherence are generally poor. Treatment switching may be considered in order to maximise benefits from pharmacological therapy, but there are relatively few data on OAB therapy switching to second and third-lines of medication. There are also few formal analyses on the impact of age, gender and choice of initial OAB drug on discontinuation rates.

**Objectives:** To investigate discontinuation rates with antimuscarinics in patients newly starting OAB therapy, with regard to patterns of switching to alternative medication, and the potential impact of age, gender and choice of initial drug.

**Methods:** Data on prescription drug use in Canada were retrieved from the IMS Brogan public and private prescription claims databases. Medication usage was tracked for four years following an index claim. The primary endpoint was the number of days from index claim to discontinuation of medication. Secondary endpoints were the number of days on first-line therapy before switching. Descriptive results were evaluated using univariate (Kaplan-Meier) and multivariate (Cox proportional hazards) models.

**Results:** Data were available for 31,754 patients. Approximately 91% discontinued OAB medication within the four-year follow-up period. The discontinuation rate was similar between men and women. The risk of discontinuation in patients ≥75 years was only slightly higher than that in patients aged 40−64 years (hazard ratio of 1.08) and was lower than in those aged 65−74 years. Retention when oxybutynin was the initial drug was lower than with most of the other antimuscarinics. Only 12.5% of patients changed OAB medication during the 4-year period. Women were more likely than men to switch from first-line or second-line treatment.

**Conclusions:** Discontinuation of initial antimuscarinic therapy was high. Compared with oxybutynin, several alternative antimuscarinics offered lower risks of discontinuation. The majority of patients had no trial of second-line treatment.

## BACKGROUND

Overactive bladder (OAB) is a chronic condition[1] which may be associated with a significant negative impact on quality of life.[2] OAB is also associated with an increased risk of falls and fractures, urinary tract and skin infections, sleep disturbance and depression.[3] Even after adjustment for age, gender and multiple comorbid conditions, urinary incontinence increases the risks of hospitalisation and admission to a nursing home.[4] OAB symptoms occur in 11.8−16.5% of adults, at a similar incidence in both women and men, and prevalence increases with age.[5] OAB will become an increasingly common problem as the number of older people in the population increases. In Canada, the proportion of people aged ≥65 years rose from 8% to 14% between 1960 and 2009, and it is estimated that this will rise to 23−25% by 2036; the population aged ≥80 years will more than double between 2009 and 2036.[6] A similar pattern is projected for the UK and the majority of other EU-27 countries.[7]

Non-drug therapies (bladder training, urgency suppression, pelvic floor muscle training, and education on bladder control strategies) are generally advocated as first-line therapy for OAB and urgency incontinence.[8] Long-term adherence to these measures requires motivation and support, and not all healthcare providers have sufficient time or resources to offer assistance to patients. Furthermore, the majority of these measures require that patients have the cognitive ability to learn new behaviours, making them a less viable option for those with cognitive impairment.

If non-drug therapies are unsuccessful, pharmacotherapy with antimuscarinic drugs is currently the mainstay of medical therapy[9], either alone or as an adjunct to behavioural therapies. Long-term medication may be required, which means that adherence and persistence are important factors affecting treatment outcomes. Adherence (the extent to which a patient acts in accordance with the prescribed interval and dose of a dosing regimen) and persistence (the time from initiation to discontinuation of therapy)[10] are typically low in many chronic diseases[11,12], but those seen with antimuscarinic drug treatments in OAB appear to be especially poor. For example, in a systematic review of medical claims studies in OAB, rates of discontinuation from antimuscarinics ranged from 43−83% within the first 30 days[13], and in a retrospective study of UK prescription data, 65−86% of patients had discontinued initial antimuscarinic treatment after 12 months.[14] In a Canadian retrospective cohort study of patients aged ≥66 years the 2-year withdrawal rates from oxybutynin (n=31,996) and tolterodine (n= 24,855) were 91% and 86%, and median times to discontinuation were 68 and 128 days, respectively.[15] Reasons for poor persistence with therapy with this group of drugs include their relative intolerability, particularly due to the most common adverse events of dry mouth and constipation, unrealistic expectations from the therapy, and perceived clinical inefficacy.[16]

Clinicians often advocate treatment switching in order to maximise benefits from pharmacological therapy, but there are relatively few data on OAB therapy switching to second and third-lines of medication. Likewise there are few formal analyses on the impact of age, gender and choice of initial OAB drug on discontinuation rates. The overall objective of this study was to examine these factors in patients newly starting on OAB therapy, using a 4-year retrospective analysis of prescription claims data from three public and private drug plans in Canada.

## PATIENTS ANS METHODS

Private and public prescription claims databases from the Canadian Private Drug Plans (PDP), Ontario Public Drug Plan (OPDP) and Régie de l'assurance maladie du Québec (RAMQ), as provided by IMS Brogan (IMS Health Canada Inc., Kirkland, Quebec, Canada) were used for this study. Ontario and Quebec are the two most populous Canadian provinces (representing approximately 62% of the Canadian population) and the IMS Brogan databases are highly representative of the Canadian population covered by public plans [Unpublished data, IMS Health Canada Inc]. The OPDP database provides prescription claims data from point-of-sale (pharmacy) or paper claim submissions and has a 100% data capture rate. The OPDP plan covers approximately 2.7 million active claimants and pays for 139 million prescriptions. RAMQ has approximately 2 million active claimants and pays for more than 170 million prescriptions. The IMS Brogan RAMQ database is a random sample of the total data, and represents about 30% of OAB prescription claims in this drug plan. The IMS Brogan PDP database is the largest national private drug plan claims database and captures approximately 70% of private claims nationally. All patients had continuous coverage throughout the monitored period.

Prescriptions for OAB drug therapies were tracked over a four-year period for treatment-naïve patients who received one of the following as starting drugs: oxybutynin (generic oxybutynin 2.5/5 mg tablets or branded oxybutynin extended release (ER) 5/10/15 mg tablets), tolterodine immediate release (IR) 1/2 mg tablets, tolterodine ER 2/4 mg capsules, solifenacin 5/10 mg tablets, darifenacin 7.5/15 mg tablets, trospium 20 mg tablets, or flavoxate generic or branded 200 mg tablets. Except for generic oxybutynin IR and generic flavoxate, all other prescription claims were for branded drugs. Data for all dosages of each index drug were grouped together. Topical formulations were not included in the analysis. Fesoterodine was not included as it was not available in Canada during the whole study period. Although flavoxate is not a typical antimuscarinic agent and is not widely used in Canada, it was included in the analysis because of its more frequent use in some other countries.

The study period consisted of four segments: the presence of an index antimuscarinic prescription filled between April 1, 2007 and March 31, 2008; confirmation of treatment-naïve status by examining the 12-month period prior to the index prescription date; tracking of claims during a 4-year period following the index claim; and a follow-up period of up to 3 months to ensure continuous coverage throughout the four-year study period and to determine therapy status at 4 years.

The primary endpoint was the number of days from index claim to discontinuation of OAB medication, measured between the date of the first claim for one of the target drugs (the index date) and the end of the day's supply for the last claim for a target drug, within the follow-up period. Discontinuation was defined as patients experiencing a gap in therapy longer than 60 days. Secondary endpoints were the number of days on first-line therapy before switching to second-line therapy, and the number of days on second-line therapy before switching to third-line therapy. 'Switch' was defined as a claim for an OAB drug that was different from the previous line of therapy (all changes in therapy were classified as switch, on the basis that patients used only one OAB medication at a time). Data from patients who died during the course of the study, or those with only one prescription claim (i.e. acute use), were excluded from the analysis.

Medication patterns were analysed by OAB drug, gender, and age categories (<20, 20−39, 40−64, 65−74, ≥75 years). Only anonymized patient-level data were analysed and, for the OPDP data, reporting of counts fewer than six were masked with a three.

According to requirements of the Health Research Ethics Board at the lead author's institution, no ethical committee review was required for a study of this nature.

### Statistical Analysis

Descriptive results were analysed (SAS Version 9.2) using univariate and multivariate models for the primary and secondary endpoints. Kaplan-Meier (univariate) survival curves were used to understand which variables showed a statistically significant difference on endpoints, regardless of any covariate influence. The logrank test was used to test the null hypothesis of no difference between populations in the probability of an event, based on the times of events; the null hypothesis was rejected if $p<0.05$.

In order to give an estimate of the size of the difference between pairs of variables (e.g. medications or age categories), multivariate analyses were performed using the Cox proportional hazards model to provide hazard ratios with 95% confidence intervals (CIs) and p-values, using oxybutynin as the reference drug (i.e. a hazard ratio of 1). Age category, gender and OAB medication were independent variables; a covariate was considered as a confounding variable if it changed the magnitude of the estimate for another independent variable by greater than 10% when removed from the model. The payer-type variable (PDP, OPDP and RAMQ) was also included but overall made no difference to the results.

## RESULTS

### Patient Distribution

Data were available for 31,754 patients. There were 47 patients with missing data on age or gender who were excluded from further analysis. There were 70% women (22,312) and 30% men (9,395); most were ≥65 years old (Figure 1). The majority started OAB therapy on oxybutynin (Table 1).

**Figure 1.** Number of Patients by Age Category and Gender (combined data plans)

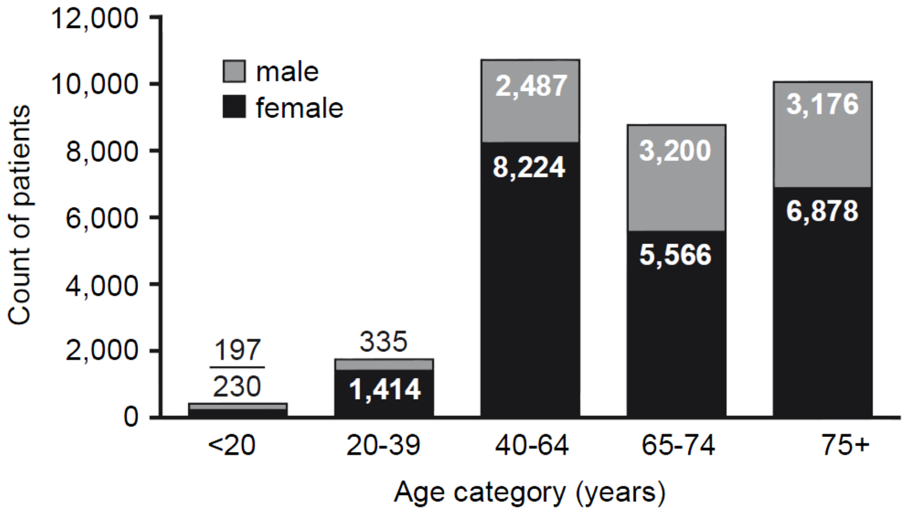

**Table 1.** Number of Patients by First OAB Medication and Insurance Plan, and Percentage Discontinuation over 4 Years (excluding patients with missing data for age or gender, n=47)

| | OXY* | SOL | TOL IR | TOL ER | DAR | TROS | FLAV | Total |
|---|---|---|---|---|---|---|---|---|
| **RAMQ, n (%)** | 3,424 (86) | 88 (2) | 91 (2) | 296 (7) | 0 (0) | 31 (<1) | 38 (<1) | 3,968 |
| **OPDP, n (%)** | 8,192 (47) | 0 (0) | 1,852 (11) | 7,561 (43) | 0 (0) | 0 (0) | 1 (<1) | 17,606 |
| **PDP, n (%)** | 4,699 (46) | 1,045 (10) | 664 (7) | 3,137 (31) | 290 (3) | 46 (<1) | 252 (2) | 10,133 |
| **Total, n (%)** | 16,315 (51) | 1,133 (4) | 2,607 (8) | 10,994 (35) | 290 (<1) | 77 (<1) | 291 (<1) | 31,707 |
| **%discontinued over 4 years** | 93 | 90 | 90 | 90 | 91 | 94 | 98 | |

OXY: oxybutynin, SOL: solifenacin, TOL: tolterodine, DAR: darifenacin, TROS: trospium, FLAV: flavoxate, IR: immediate release, ER: extended release, RAMQ: Régie de l'assurance maladie du Québec, OPDP: Ontario Public Drug Plan, PDP: Private Drug Plans

* In the RAMQ and OPDP, the oxybutynin cohort consisted of 90–94% generic oxybutynin IR and 6–10% branded oxybutynin ER (varying according to the follow-up year). In the PDP, 36–46% of patients in the oxybutynin group received branded oxybutynin ER.

## Discontinuation

Approximately 91% of patients (n=28,996) discontinued OAB medication by the end of 4 years, ranging from 90–98% between the different drugs (Table 1). The discontinuation rate was similar between men and women (91.4% vs. 91.5%, respectively). Patients ≥40 years old had a lower discontinuation rate than those aged 20–39 years (91–92% vs. 94–97%, respectively).

Therapy retention rates according to the first-prescribed drug are shown in Figure 2. Fewer than 40% of patients remained on OAB therapy after 6 months, regardless of the initial drug prescribed (ranging from 39.9% for solifenacin to 8.6% for flavoxate), and the retention rate fell to below 28% for all drugs after one year (ranging from 27.8% for solifenacin to 5.8% for flavoxate). Retention when oxybutynin was the initial drug was lower than with other antimuscarinics at all timepoints, with the exception of trospium at years 3 and 4, and flavoxate at all timepoints.

**Figure 2.** Percentage of Patients Remaining on OAB Therapy, according to First-prescribed OAB Drug

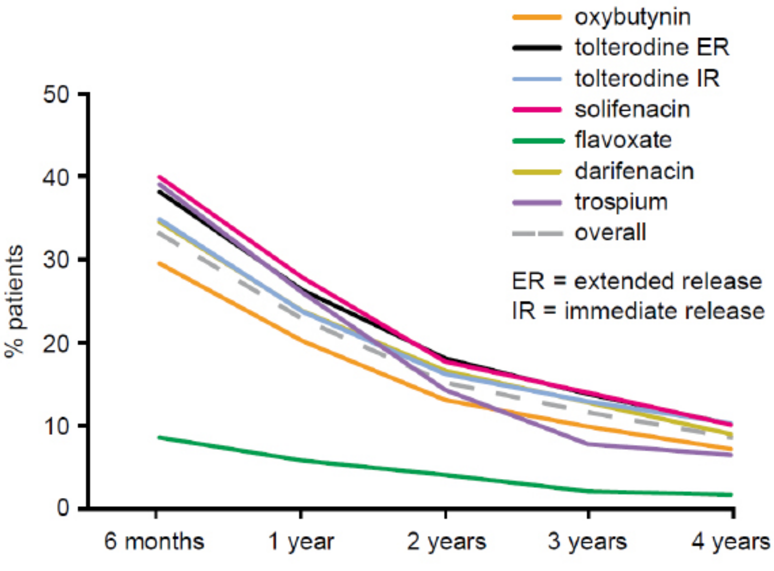

## Time to Discontinuation

The median number of days to discontinuation by age category and gender are shown in Table 2. The risk of discontinuing was 3% higher in men than women (p<0.04). Patients aged 40−64 years were at lower risk of discontinuing therapy compared with other age categories (p<0.003).

The duration of first-line treatment was as follows (median days [mean; SE]): solifenacin (106 [380; 16.1]), tolterodine ER (100 [376; 5.3]) tolterodine IR (90 [343: 10.5]), darifenacin (90 [341; 30.3]) trospium (90 [304: 48.6] oxybutynin (60 [287; 3.8]) and flavoxate (10 [83; 13.2]). In the Cox proportional hazards model, patients receiving initial treatment with solifenacin, darifenacin, tolterodine ER and tolterodine IR were at significantly lower risk of discontinuing compared with those receiving oxybutynin as the first medication (hazard ratios of 0.68, 0.72, 0.77 and 0.84, respectively [vs. 1.00 for oxybutynin], each p<0.001 vs. oxybutynin) (Figure 3). Patients receiving flavoxate as initial treatment were at significantly higher risk of discontinuing compared with those who received oxybutynin (hazard ratio 2.48, p<0.0001). There was no statistically significant difference in the risk of discontinuing OAB treatment when using trospium as first-line compared with oxybutynin (p=0.1074).

**Table 2.** Median Days on Therapy and Hazard Ratios for Risk of Discontinuation, According to Gender and Age Categories

| | n | Median Days on Therapy | Hazard Ratio | 95% CI | p-value |
|---|---|---|---|---|---|
| **Gender** | | | | | |
| male | 9,395 | 87 | 1.03 | 1.00–1.06 | 0.0341 |
| female* | 22,312 | 84 | 1.00 | | |
| **Age category (years)** | | | | | |
| 20 | 427 | 62 | 1.17 | 1.06−1.29 | 0.0022 |
| 20−39 | 1,749 | 59 | 1.19 | 1.13–1.26 | <0.001 |
| 40−64* | 10,711 | 84 | 1.00 | | |
| 65−74 | 8,766 | 85 | 1.12 | 1.08–1.16 | <0.001 |
| ≥75 | 10,054 | 90 | 1.08 | 1.04–1.11 | <0.001 |

*Reference variable, CI: Confidence Interval, n: number of patients

Survival curves for time to discontinuation in the Cox proportional hazards model are shown in Figure 4, while those from the Kaplan-Meier analysis are provided as Supplementary Information (Figure S1).

## Time to First Switch of Medications

Of all patients, 3,967 (12.5%) changed OAB medication at least once during the observation period. Patients whose initial therapy was tolterodine ER, solifenacin or darifenacin were statistically significantly less likely to switch treatments than were those who started on oxybutynin. There was no significant difference in the risk of switching for those who started on tolterodine IR, trospium or flavoxate compared to oxybutynin. Women were 20% more likely than men to switch from first-line treatment. There was no significant difference between age categories in the risk of switching, other than a lower risk for patients <20 years old (Table 3).

**Figure 3.** Hazard Ratios for Risk of Discontinuation According to First-prescribed OAB Drug

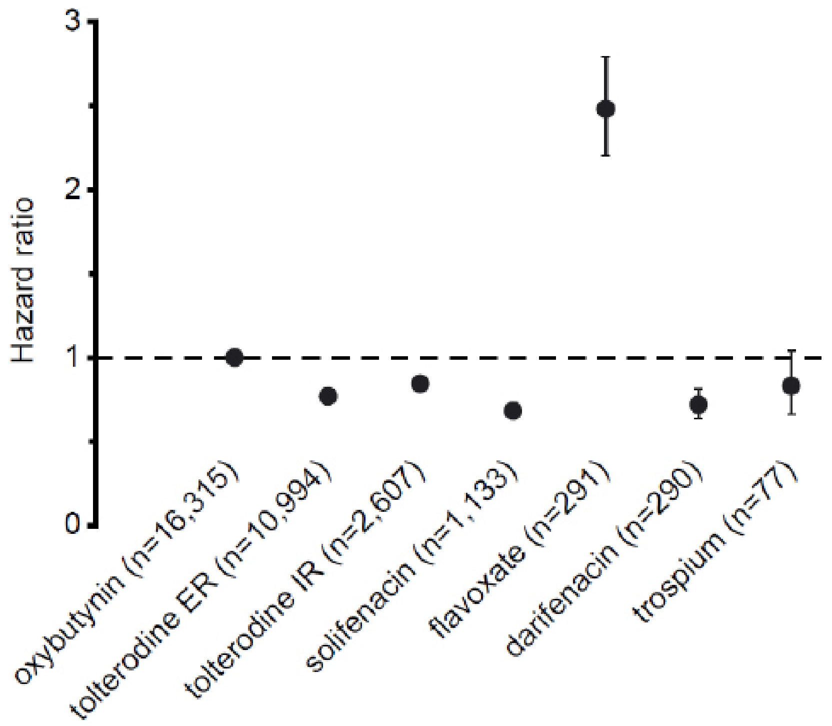

Hazard ratios (±95% CI) calculated by Cox proportional hazards model. Oxybutynin was used as the reference medication (hazard ratio = 1). Significantly lower risk ($p<0.001$) for tolterodine IR, tolterodine ER, solifenacin and darifenacin vs oxybutynin. Significantly higher risk ($p<0.0001$) for flavoxate vs oxybutynin. No statistically significant difference in risk for trospium vs. oxybutynin ($p=0.1074$); ER: extended release; IR: immediate release

Survival curves from the Cox proportional hazards model are shown in Figure 4, while those from the Kaplan-Meier analysis are provided as Supplementary Information (Figure S2).

### Time to Second Switch of Medications

Only 470 patients (1.5%) changed medications twice, representing 11.8% of the cohort who switched therapies. Patients taking solifenacin as second-line medication were statistically significantly less likely to switch to a third-line OAB therapy compared with those who received oxybutynin (hazard ratio 0.57 vs. oxybutynin 1.00, $p=0.0083$) (Table 3). Patients taking tolterodine IR or flavoxate as second-line treatment had a significantly higher risk of switching to another treatment compared with oxybutynin. There were no other significant differences in risk of switching compared with those receiving oxybutynin as second-line. Men were 22% less likely than women to switch to a third-line of therapy. Age was not a significant factor influencing the risk of switching.

Survival curves from the Cox proportional hazards model and the Kaplan-Meier analysis are provided as Supplementary Information (Figures S3 and S4).

**Table 3.** Median Number of Days on First-line and Second-line Therapy and the Hazard Ratio for Risk of Switching, according to Antimuscarinic, Gender and Age Categories

| | Univariate Analysis | Multivariate Analysis | | |
|---|---|---|---|---|
| | **Median (days)†** | **Hazard Ratio** | **95% CI** | **p-value** |
| **First-line Medication** | | | | |
| oxybutynin (n=2,509) | 60 | 1.00 | * | * |
| tolterodine ER (n=858) | 136 | 0.39 | 0.36−0.43 | <0.0001 |
| tolterodine IR (n=387) | 98 | 0.90 | 0.81−1.01 | 0.0655 |
| solifenacin (n=147) | 90 | 0.56 | 0.47−0.66 | <0.0001 |
| darifenacin (n=35) | 86 | 0.55 | 0.39−0.77 | 0.0005 |
| flavoxate (n=18) | 80 | 0.94 | 0.59−1.49 | 0.7775 |
| trospium (n=13) | 70 | 0.79 | 0.46−1.36 | 0.3926 |
| **Gender** | | | | |
| male (n=997) | 86 | 0.80 | 0.75−0.86 | <0.0001 |
| female (n=2,970) | 72 | 1.00 | * | * |
| **Age category (years)** | | | | |
| <20 (n=20) | 104 | 0.33 | 0.21−0.51 | <0.0001 |
| 20−39 (n=187) | 74 | 0.94 | 0.81−1.09 | 0.4204 |
| 40−64 (n=1,420) | 80 | 1.00 | * | * |
| 65−74 (n=1,010) | 68 | 0.99 | 0.90−1.08 | 0.8138 |
| ≥75 (n=1,330) | 77 | 1.05 | 0.96−1.14 | 0.2872 |
| **Second-line Medication** | | | | |
| tolterodine ER (n=216) | 216 | 0.76 | 0.51−1.12 | 0.1621 |
| tolterodine IR (n=94) | 142 | 1.93 | 1.29−2.90 | 0.0014 |
| solifenacin (n=77) | 198 | 0.57 | 0.37−0.86 | 0.0083 |
| oxybutynin (n=43) | 126 | 1.00 | * | * |
| darifenacin (n=20) | 85 | 1.38 | 0.80−2.39 | 0.2502 |
| trospium (n=14) | 60 | 1.423 | 0.76−2.66 | 0.2697 |
| flavoxate (n=6) | 60 | 4.73 | 1.98−11.32 | 0.0005 |
| **Gender** | | | | |
| male (n=99) | 264 | 0.78 | 0.62−0.98 | 0.0339 |
| female (n=371) | 144 | 1.00 | * | * |
| **Age category (years)** | | | | |
| <20 (n=1) | 28 | 0.50 | 0.07−3.55 | 0.4837 |
| 20−39 (n=25) | 91 | 0.88 | 0.57−1.35 | 0.5609 |
| 40−64 (n=219) | 130 | 1.00 | * | * |
| 65−74 (n=107) | 188 | 1.07 | 0.82−1.39 | 0.6421 |
| ≥75 (n=118) | 289 | 0.96 | 0.73−1.25 | 0.7392 |

*Reference variable; † Logrank p<0.001 for all variates; n: number of patients who switched; IR: immediate release; ER: extended release; CI: confidence interval

**Figure 4.** Time to Discontinuation and First Switch (Cox proportional hazards model)

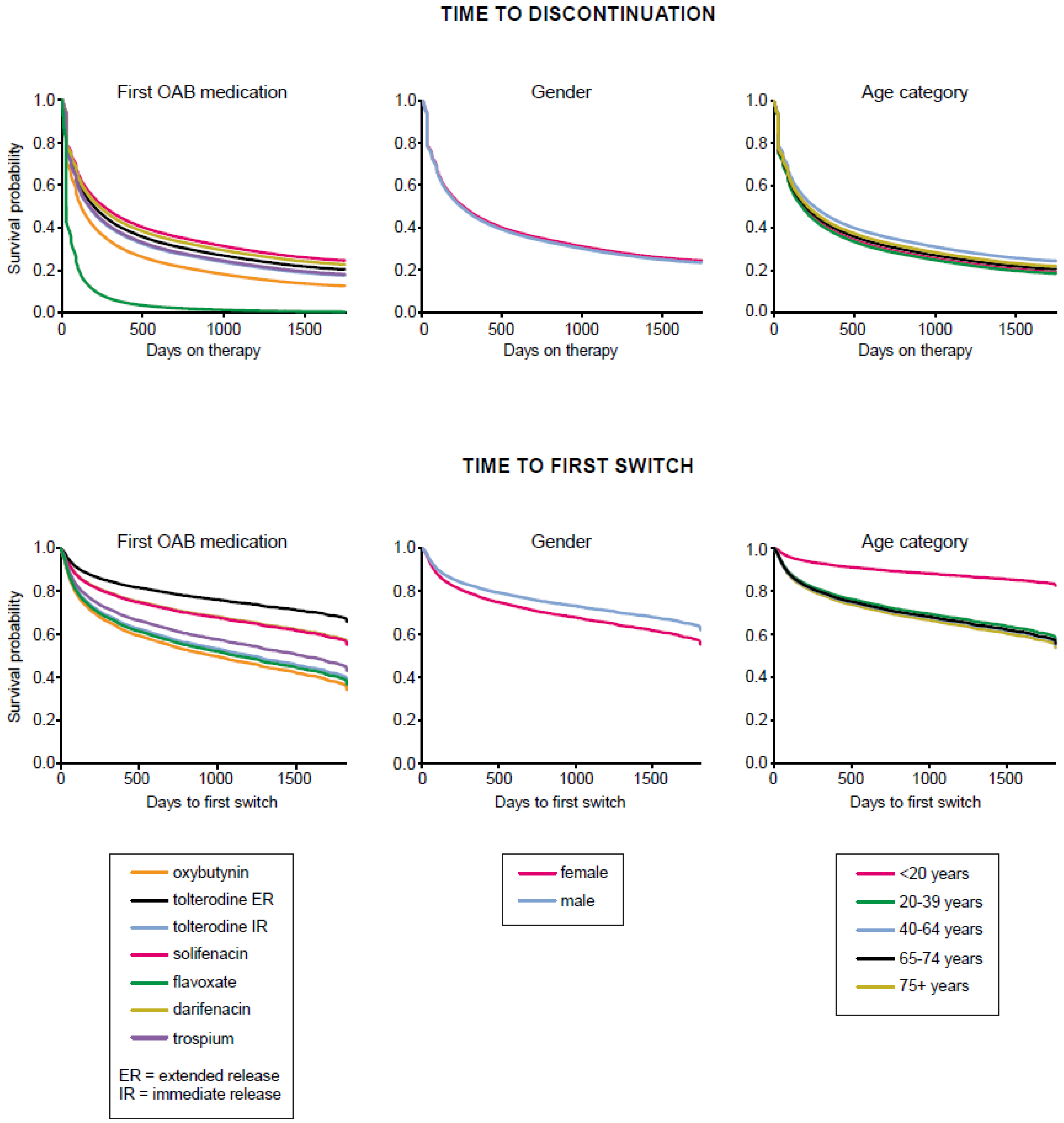

ER: extended release; IR: immediate release

## DISCUSSION

OAB represents a challenge in terms of effective long-term management and adequate provision of healthcare resources. This is particularly important for older people, who, as a proportion of the population will increase with time, have a greater risk of OAB-associated adverse events, and are more likely than younger people to receive inadequate treatment for urinary incontinence.[17] In the elderly, antimuscarinics are effective and their use does not appear to be associated with an increased risk of falls or delirium.[18,19]

The distribution of patients receiving drug treatment in this study mirrors the age-related distribution in epidemiological studies[5], with relatively few patients (<7%) being under 40 years old and a high proportion (>30%) aged 75 years or older. In contrast to epidemiological studies showing a small difference between men and women in the prevalence of OAB symptoms, in this study female claimants outnumbered men by approximately 2:1. This might reflect the reluctance of clinicians to treat men with antimuscarinics in the belief that LUTS in men are predominantly prostatic in origin, and for the often unfounded fear of inducing acute painful retention.[20]

The four-year discontinuation rate of 91% and a median of 60−106 days on the various drugs before discontinuation (excluding flavoxate) were generally consistent with other studies. Any differences might be due to the different methods used for data collection and analysis, and in the way persistence was defined. Although discontinuation was high with all the antimuscarinics, flavoxate (a non-antimuscarinic) performed especially poorly for all endpoints. Patients receiving solifenacin, darifenacin, tolterodine ER or tolterodine IR as initial treatment were significantly less likely to discontinue therapy than were those who received oxybutynin as the first-line medication.

There was a significantly lower risk of discontinuation of first-line therapy in patients aged 40−64 years compared with other age categories in the multivariate model. It is often assumed that prescribers might stop medication in patients over 80 years of age because of general concerns about giving antimuscarinics to this age group, or a perceived diminution of the importance of OAB compared with other comorbid conditions, or a higher rate of adverse effects.[14] Our data did not support this assumption, as the risk of discontinuation in patients ≥75 years was only slightly higher than that in patients aged 40−64 years and was lower than in those aged 65−74 years. Men were slightly more likely than women to discontinue OAB therapy during the four-year study period, but this is of limited clinical significance, other than to reinforce the view that antimuscarinics are equally well accepted by men and women.

Other studies have shown that if an antimuscarinic is ineffective it may be beneficial to switch to an alternative agent.[21-23] For example, patients dissatisfied with previous extended release antimuscarinic therapy experienced efficacy and a worthwhile increase in total treatment duration by switching to darifenacin[21] and fesoterodine.[22] Similarly, an open-label study found that patients with insufficient subjective improvement in urgency with tolterodine ER 4 mg had significantly reduced OAB symptoms and improvements in HRQL and social interaction when switched to solifenacin 5/10 mg.[23] However, in a Norwegian study of prescription data, overall 1-year persistence for new users was 38.0%, but only 10.3% switched from their initial antimuscarinic, and 51.7% discontinued without switching.[24] The majority of patients in our study also received just one line of treatment, with only 12.5% switching therapies, and only 1.5% receiving three lines of medication. By not being offered a change of therapy, this raises concerns about under-treatment and loss to care, or to unnecessarily early escalation of therapy to more invasive options. This may reflect Canadian prescribing guidelines, drug availability and drug costs: in the majority of Provinces, funding criteria are established for the patient to receive oxybutynin as first-line treatment (other than flavoxate, oxybutynin is the only generic OAB drug available in Canada), and patients only become eligible for use of branded antimuscarinics after failure of an adequate trial of oxybutynin.

Patients who received solifenacin, darifenacin or tolterodine ER as initial treatment were significantly less likely to switch treatments than those who started on oxybutynin. For patients who switched to a third-line of medication, only those who received solifenacin as second-line medication were statistically significantly less likely to switch compared to when oxybutynin was given as second-line, but the number of patients here was small and conclusions should be made with caution. There were no significant differences between age categories in the risk of discontinuation in patients who switched to second or third-line medication. Women were about 20% more likely than men to receive second or third-line OAB medication, suggesting that men were lost to treatment.

### Study Limitations

There are several limitations to this analysis. There was no information available on the reasons for prescription or the speciality of the doctor who prescribed the medication (medical records and diagnosis information were unavailable), but we consider it unlikely that there was significant use of the target OAB drugs for long-term treatment of diagnoses other than OAB. Likewise we can ascribe no cause to the cessation of therapy for any patient, whether this was patient or physician initiated, whether purposeful or in response to inadequate efficacy or intolerability of the previously prescribed drug. The study protocol focused only on antimuscarinic drugs that were available in Canada during the 4-year study period and, for example, it was not possible to include data on fesoterodine. There was no information on individual doses prescribed or dose adjustments. It is possible that some patients might have discontinued therapy because they achieved continence or another satisfactory outcome. The identification of 'as needed' therapy or 'drug holidays' of less than the 60-day grace period was not possible.

The pre-specified protocol for the analysis grouped together the data from all formulations of oxybutynin (generic IR and branded ER), on the basis that branded ER formulations have a relatively small share of the OAB market in Canada. On completion of the analysis it became evident that prescription claims for oxybutynin ER were higher than anticipated (up to 10% in the public plans, but up to 46% in the private plan). A Canadian study found that persistence with oxybutynin ER was higher than with IR after 12 months (18.9% vs. 13.8%, respectively) [Unpublished; personal communication: Wagg, A] and this was also observed in a 12-month study of UK prescription data which used different methods for measuring persistence (26% vs. 22% persistence with ER and IR, respectively).[14] On this basis, including results for oxybutynin ER within an overall 'oxybutynin' grouping in our study would have underestimated the discontinuation rate with oxybutynin IR.

Finally, there was no information on how much support might have been offered to patients by clinicians, patient support groups, continence advisors and others. Medication support programmes have been shown to positively influence persistence with treatment.[25,26]

## CONCLUSION

Discontinuation of initial antimuscarinic therapy in OAB is high, and presents a continuing therapeutic challenge. The most widely used first-line antimuscarinic in this study was oxybutynin, which benefits from being the lowest cost option and is funded in all provincial drug plans across Canada, as well as in many other countries and jurisdictions. Although economic factors make it unlikely that this situation will change radically, this study has shown that alternative antimuscarinics may offer significantly lower risks of discontinuation. The majority of patients received only one line of treatment, and had no second-line treatment trial. The concept of switching therapies to offer patients an optimized individual approach needs further exploration.

## ACKNOWLEDGEMENTS

Editorial assistance was provided by Steven Sharpe, PhD of SharpeCom Ltd (Midhurst, West Sussex, UK), and was funded by Astellas Pharma Canada Inc.

Retrospective prescription claims data and statistical analyses were provided by IMS Brogan (IMS Health Canada Inc., Kirkland, Quebec, Canada): Team members: Kristen Reidel (Statistician), Brad Millson (Engagement Manager), Nevzeta Bosnic (Principal), Kimberley Tran (Consultant). The statements, findings, conclusions, views, and opinions contained and expressed in this article are based in part on data obtained under license from the following IMS Brogan information service(s): OAB LOT Study. Data Period 2007-2014. All Rights Reserved. The statements, findings, conclusions, views, and opinions contained and expressed herein are not necessarily those of IMS Brogan or any of its affiliated or subsidiary entities.

### Conflict of Interest Declaration

This study was funded by Astellas Pharma Canada, Inc. AW is either speaker, researcher or consultant for Astellas Pharma, Merus Labs, Pfizer Corp, Svenska Cellulosa Aktiebolaget. DD is an employee of Astellas Pharma Canada, Inc. TB was formerly an employee of Astellas Scientific & Medical Affairs, Inc.
